# Influence of Baromi-2 Rice Flour Particle Size on Gluten-Free Batter Rheology and Quality Characteristics of Deep-Fat Fried Chicken

**DOI:** 10.3390/foods14162836

**Published:** 2025-08-15

**Authors:** Dajeong Oh, Yi Ho Jeon, Youngjae Cho

**Affiliations:** 1 Department of Food Science and Technology, Pusan National University, Miryang 50463, Republic of Korea; oh9906@naver.com (D.O.); jih2792@naver.com (Y.H.J.); 2 Food Tech Innovation Center, Pusan National University, Miryang 50463, Republic of Korea

**Keywords:** Baromi-2, rice, batter, fried chicken, particle size

## Abstract

With the rising trend of health-conscious consumers, demand for gluten-free alternatives is increasing, and rice flour is a promising gluten-free alternative for chicken batter. This study examines the effects of particle size variations in Baromi-2 rice flour on batter rheology and the quality attributes of deep-fat fried chicken. Baromi-2 is a rice variety specifically developed to meet the demands of the modern food processing industry, especially for applications requiring dry milling. Five particle sizes (60, 100, 120, 160, and 180 mesh) were evaluated on the basis of their physicochemical properties, including water-holding capacity (WHC), amylose content, and damaged starch levels. Batter consistency was assessed and frying performance was analyzed with regard to coating pickup, cooking loss, moisture content, crust color, and textural attributes. Results demonstrated that finer particle sizes (e.g., 180 mesh) exhibited high WHC and batter viscosity, resulting in reduced flowability and enhanced adhesion. These properties contributed to high coating pickup, improved moisture retention, and reduced cooking loss during frying. Fried chicken prepared with finer particles showed soft textures, great cohesiveness, and light crust colors with high lightness (L*) and reduced redness (a*) and yellowness (b*), producing a visually appealing product. By contrast, larger particle sizes (e.g., 60 mesh) resulted in low viscosity, uneven coatings, and high cooking loss. This study highlights the critical role of rice flour particle size in optimizing batter functionality and improving the quality of fried foods. Furthermore, these findings suggest the potential to bridge the gap between consumer demand for healthier fried foods and the food industry’s demands.

## 1. Introduction

Deep-fat frying is widely used to enhance the sensory appeal of food by imparting a crispy texture, rich flavor, and a golden-brown crust. This method involves simultaneous heat and mass transfer, where water is evaporated, and oil is absorbed into the food, creating its characteristic texture and structure. However, excessive oil absorption during frying raises health concerns, prompting research into methods to optimize batter formulations and frying conditions to reduce oil uptake without compromising quality [1,2].

Batter composition plays a pivotal role in determining the sensory and structural properties of fried products. Recent studies have highlighted the potential application of rice flour as a gluten-free alternative to wheat flour, providing hypoallergenic properties, digestibility, and functional versatility. Notably, rice flour-based batters have been shown to exhibit superior crispness and lower oil absorption compared with their wheat-based counterparts. This difference is attributed to the physicochemical properties of rice flour, including its amylose content, viscosity, and particle size. A higher amylose content is associated with enhanced crispness and reduced oil uptake, and smaller particle sizes improve batter adhesion, water absorption, and textural properties [3,4,5].

Compared with wheat, rice exhibits a unique starch structure and lower gluten content, which significantly influences batter rheology and frying performance. For example, rice flour batters have demonstrated superior coating properties and low oil uptake because of their ability to form compact and stable starch networks during frying. The reduced oil absorption and improved sensory properties of rice flour make it advantageous for health-conscious consumers [4,6]. Additionally, rice flour used in chicken batter has shown promise as a gluten-free substitute for wheat flour, providing a solution for those with celiac disease or gluten sensitivity [7].

Baromi-2 used in this study is a rice variety specifically developed to meet the demands of the modern food processing industry, especially for applications requiring dry milling. This early-maturing variety, created by crossing ‘Suweon542’ with ‘Jopyeong’, was bred to have low grain hardness, which makes it ideal for milling without the need for pre-treatment processes like soaking or steeping [8].

Chicken breast, a lean protein source with high nutritional value, is widely used in fried food products because of its high-protein and low-fat content, making it suitable for health-conscious diets. However, the use of rice flour-based batters in chicken frying presents difficulty in achieving optimal adhesion, crispness, and moisture retention. Previous studies have highlighted that batter properties, including particle size and amylose-to-amylopectin ratios, significantly influence the oil uptake, texture, and sensory characteristics of fried chicken products [5,9].

Several studies have explored the impact of rice flour particle size on batter functionality and frying performance. Small particle sizes enhance the hydration capacity and viscosity of batters, thereby improving the pickup ratio and adhesion to food surfaces. Furthermore, small particles facilitate the formation of thin, uniform coatings that effectively limit oil absorption and contribute to a crisp texture after frying. For example, Matsunaga et al. [4] demonstrated that starch granule size and gelatinization properties influence the crispness and water retention of tempura batters. Similarly, Nakamura and Ohtsubo [5] found that rice flour with a high-amylose content and reduced particle size significantly lowers oil uptake while maintaining desirable sensory properties.

Despite these findings, a notable research gap exists in understanding the mechanism by which rice flour particle size affects the rheological properties of batters and the quality of deep-fat fried chicken. In addition, varieties such as Baromi-2, which is known for its unique starch structure and reduced damaged starch content, have not been extensively studied in this context. Baromi-2 exhibits physical properties that are comparable to wheat flour, such as its low gelatinization temperature and compact starch granules, which enhance batter performance during frying [8].

This study aims to investigate the influence of particle size variations in Baromi-2 rice flour on batter rheology and the quality characteristics of deep-fat fried chicken breast. It also aims to provide a comprehensive understanding of the mechanism by which rice flour particle size affects batter functionality and fried product quality by examining key factors such as oil absorption, texture, and moisture retention. The findings of this study will provide insights into the development of healthier, high-quality fried foods while expanding the commercial application of rice flour-based batters in the food industry.

## 2. Materials and Methods

### 2.1. Materials

The processed rice used in this study, Baromi-2, was purchased from Serom Food (Icheon, Republic of Korea). The wheat flour (Beksul^®^ soft flour, CJ CheilJedang, Yangsan, Republic of Korea) was purchased at a local store. The Starch Damage Assay Kit was obtained from Megazyme International Ireland, Ltd. (Wicklow, Ireland). All chemicals used in the study were of analytical grade.

### 2.2. Preparation of Rice Flour

Rice flour was produced by dry milling using a blade mill (HKB-100, KPS, Incheon, Republic of Korea). The milled rice flour was subsequently sieved through mesh screens of varying sizes (60, 80, 100, 120, and 160 mesh) to obtain different particle size distributions.

### 2.3. Particle Size Distribution

The particle size distribution and mean particle size of the rice flour samples were determined using a particle size analyzer (LS 13 320, Beckman Coulter, Brea, CA, USA) in the powder mode [10].

### 2.4. Moisture Content of Rice Flour

Moisture content was assessed by drying 1 g of rice flour in a forced convection oven (OF4-15SW, Lab Companion, Seoul, Republic of Korea) at 105 °C for 24 h or until a constant weight was achieved, in accordance with the AOAC guidelines [11].

### 2.5. Water-Holding Capacity (WHC)

Water-holding capacity (WHC) was determined by centrifugation [12]. Briefly, 1 g of rice flour was combined with 20 mL of distilled water in a conical tube and vortexed for 10 s. The mixture was incubated in a shaking incubator at 30 °C for 25 min, followed by centrifugation at 5000× *g* for 10 min. The supernatant was carefully removed, and the WHC was calculated using the following equation:

WHC(%) = ((water absorbed weight − initial sample weight)/initial sample weight) × 100

### 2.6. Amylose Content

Amylose content was quantified using a modified method described by Jain et al. [13]. A standard curve was constructed using potato amylose (A0512-1G, Sigma Aldrich Chemical Co., St. Louis, MO, USA). A 40 mg sample of amylose was dissolved in 1 mL of 95% ethanol and 9 mL of 1 N NaOH, heated in a boiling water bath for 10 min, and diluted to 100 mL with distilled water. Rice flour samples (100 mg) were prepared following the same procedure. A 5 mL aliquot of the prepared rice solution was transferred to a volumetric flask, to which 1 mL of 1 N acetic acid and 2 mL of iodine-potassium iodide solution were added. The solution was diluted to 100 mL, mixed thoroughly, and incubated for 20 min. The absorbance at 620 nm was measured using a colorimeter. Amylose content was calculated based on the standard curve.

### 2.7. Damaged Starch

Damaged starch content was determined using the Starch Damage Assay Kit (KSDAM, Megazyme International, Wicklow, Ireland) following AACC method 76-31.01.

### 2.8. Color of Rice Flour

The color attributes (L*, a*, b*) of rice flour samples were measured using a colorimeter (CR-400, Konica Minolta Inc., Tokyo, Japan) [14]. Measurements were repeated five times per sample, and the mean values were recorded. Calibration was performed using a white standard plate (x = 86.3, y = 3161, z = 3216).

### 2.9. Pasting Properties

The pasting properties of rice flour samples were evaluated using a Rapid Visco Analyzer (RVA 4500; Perten Instruments, Sydney, Australia) [15]. A 3 g rice flour sample was mixed with 25 g of distilled water in the RVA canister. The measurement protocol involved an initial holding temperature of 50 °C for 1 min, followed by heating to 95 °C and holding for 2 min and 30 s. After the heating phase, the sample was cooled to 50 °C and held for an additional 1 min and 30 s. The parameters recorded from the viscogram included peak viscosity, trough viscosity, final viscosity, setback, breakdown, and pasting temperature.

### 2.10. Textural Properties

The textural properties of rice flour were evaluated using a texture analyzer (CT3, Brookfield, Middleboro, MA, USA) in the texture profile analysis (TPA) mode [16]. Rice flour gels (15%) were prepared by mixing 10 g of rice flour with water, stirring at 35 °C for 1 min, and heating at 95 °C for 30 min. The gels were subsequently cooled overnight at 4 °C and then set to 25 °C for analysis. Measurements were conducted using a P/5 probe with a test speed of 1 mm/s, a compression distance of 10 mm, and a compression interval of 5 s.

### 2.11. Preparation of Fried Chicken Samples

Batter formulations were prepared by mixing 100 g of rice flour, 3 g of sodium chloride, and 1 g of sodium bicarbonate with 140 g of distilled water. The mixture was continuously stirred for 5 min to ensure homogeneity. A wheat flour-based batter formulation was prepared using the same ratio for comparative analysis.

Refrigerated chicken breast, purchased one day prior to the experiment, was used to maintain freshness. The chicken was uniformly cut into 3 cm × 3 cm × 2 cm pieces (width × length × height) as described by Alugwu et al. [17] to ensure consistency in experimental trials. The frying process was conducted using a deep fryer at 170 °C for 3 min and 30 s. Fried samples were cooled to 40 °C at room temperature prior to physicochemical property assessments and texture analysis.

### 2.12. Pick-Up and Cooking Loss

Pick-up, defined as the amount of batter adhered to the chicken, was assessed by dipping chicken pieces into the batter, holding them for 5 s to allow excess batter to drip, and weighing them before frying. Cooking loss was determined by measuring the weight difference before and after frying [18].

$$\mathrm{Pick}\text{-}\mathrm{up}\;(\%)=\frac{\left(B-I\right)}{I}\times 100$$

$$\mathrm{cooking}\;\mathrm{loss}\;(\%)=\frac{\left(B-F\right)}{I}\times 100$$

where B represents the battered weight, I is the initial weight, and F is the final weight.

### 2.13. Moisture Content of Fried Chicken

The moisture content of the fried chicken samples was determined using the oven-drying method outlined by the AOAC official method [19]. Fried samples were weighed immediately after frying and cooling. Each sample was then placed in a drying oven set at 105 °C and dried for 48 h until a constant weight was obtained. Following the drying process, the samples were stabilized in a desiccator for 1 h before recording their final weight.

### 2.14. Texture Profile Analysis of Fried Chicken

The texture profile analysis (TPA) of fried chicken samples was conducted using a texture analyzer (CT3, Brookfield, Middleboro, MA, USA) equipped with a TA4/1000 cylindrical probe (38.1 mm diameter) [20]. The analysis parameters included a test speed of 5 mm/s, a compression distance of 10 mm, a triggering load of 10 g, and a compression interval of 5 s. These conditions were set to ensure the precise measurement of textural properties such as hardness, cohesiveness, springiness, and chewiness, providing a comprehensive assessment of the texture characteristics of the fried chicken.

### 2.15. Statistical Analysis

Statistical analyses were performed using SPSS software (version 26, SPSS Inc., Chicago, IL, USA). All measurements were conducted in triplicate, and the results were expressed as means with standard deviations. One-way analysis of variance (ANOVA) was performed to determine significant differences among the samples. Duncan’s multiple range test was applied to identify statistically significant differences between sample groups at a significance level of *p* < 0.05.

## 3. Results and Discussion

### 3.1. Particle-Size Distribution of Rice Flour

The particle-size distribution of rice flour is a critical factor influencing its physicochemical properties and processing quality, thereby affecting the edibility and sensory characteristics of the final product [21]. Table 1 shows the particle-size distribution of rice flour produced by dry milling and classified using mesh sizes of 60, 100, 120, 160, and 180. The data demonstrate a clear trend: decreasing mesh sizes result in a corresponding decrease in mean particle size and specific percentiles (D10, D50, and D90). For example, the mean particle size of rice flour passed through a 60-mesh sieve was 88.57 µm, whereas that passed through a 180-mesh sieve was 32.69 µm. This pattern was consistently observed across all the measured percentiles, indicating a uniform and fine particle-size distribution with smaller mesh sizes. By contrast, the wheat flour sample exhibited a mean particle size of 43.96 µm, which is intermediate between that of the 100 and 120-mesh rice flour samples. The D10 and D90 values of wheat flour are 7.80 and 104.35 µm, respectively, indicating a broader particle-size distribution compared with rice flour processed through finer mesh sizes.

**Figure 1:**
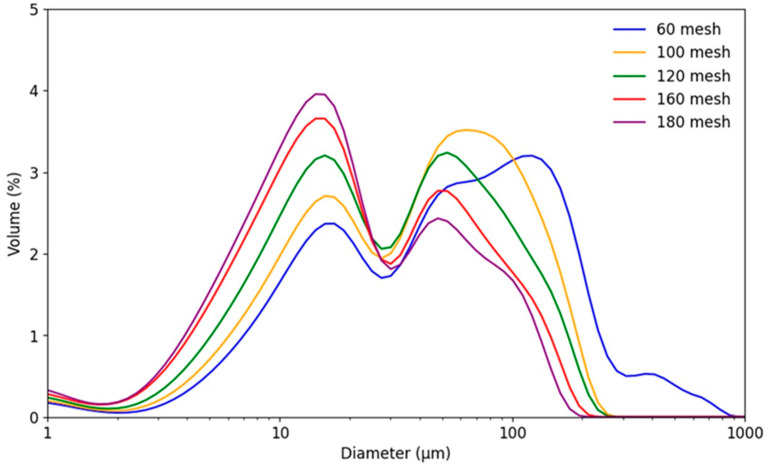
Particle-size distribution of rice flour with different mesh sizes.

The bimodal distribution of rice flour particles can be attributed to the physical effects of milling and the inherent structure of starch and protein in rice (Figure 1). In a previous study, small particles were primarily composed of starch granules that were separated from the protein matrix, whereas large particles contained starch granules that were still partially attached to the protein matrix [22]. This variation leads to a spectrum of particle sizes in the production of rice flour. In addition, small particles tend to have high levels of damaged starch, low viscosity, and increased digestibility. These characteristics enhance the digestive efficiency, texture, and appearance of the final products. Consequently, particle-size distribution significantly influences the functional properties of rice flour, making it a crucial parameter for management during milling.

### 3.2. Physicochemical Properties of Rice Flour

The impact of particle size on the physicochemical properties of rice flour was analyzed. The moisture content of rice flour significantly decreased with the decrease in particle size, with values declining from 10.66 ± 0.94% for 60-mesh samples to 5.54 ± 0.43% for 180-mesh samples (Table 2). This trend can be attributed to the increased surface area of small particles, which enhances evaporation efficiency during milling and storage.

The water-holding capacity (WHC) of rice flour significantly increased with the decrease in particle size, with values increasing from 1.11 ± 0.02% for 60-mesh samples to 1.36 ± 0.02% for 180-mesh samples. This trend is due to the great surface area and porosity of small particles, thereby improving water retention [12]. Such an increase in WHC indicates the potential application of rice flour in the food industry that requires high hydration, such as gluten-free baked products [23].

Amylose content slightly increased with the decrease in particle size, ranging from 20.33 ± 0.16% in the 60-mesh samples to 21.67 ± 0.23% in the 180-mesh samples (Table 2). This trend may be attributed to the disruption of starch granules during milling, exposing more amylose in finer particles. Amylose has a linear structure and can easily form hydrogen bonds with water to form a gel network, which greatly contributes to the increase in the viscosity of starch gels [24]. Therefore, as the amylose content increases, the viscosity of the dough increases, which in turn makes the internal gel structure denser and reduces the oil absorption [25]. These characteristics can be utilized when trying to suppress oil absorption during the frying of rice flour-based dough products [26].

Damaged starch content also increased from 4.45 ± 0.06% for 60-mesh samples to 9.21 ± 0.01% for 180-mesh samples. This increase may be due to the great mechanical force applied during fine milling, which disrupts starch granules and leads to a high level of damaged starch. As the damaged starch content increases, the elastic modulus (G′), viscous modulus (G″) and agglomeration of starch granules of rice flour increase, but the peak viscosity, breakdown value, and enthalpy change (ΔH) decrease [27]. Rice flour with high damaged starch content not only has low gelatinization stability and easy retrogradation, but also increases water binding due to the hydration reaction between damaged starch and water, resulting in a hard dough texture [28]. Therefore, an appropriate amount of damaged starch is beneficial to the production of high-quality foods, but a high content of damaged starch may harm food quality [29].

Regarding color properties, the lightness (L*) value increased with the decrease in particle size, ranging from 92.96 ± 0.03 for 60-mesh samples to 94.23 ± 0.02 for 180-mesh samples. This increase in lightness is attributed to the enhanced light scattering of fine particles [28]. The redness (a*) value ranged from −0.090 ± 0.01 to −0.057 ± 0.00, with all samples displaying a slight tendency toward the green spectrum. The yellowness (b*) value decreased with finer particle sizes, dropping from 5.24 ± 0.01 for 60-mesh samples to 4.14 ± 0.03 for 180-mesh samples. This reduction may be attributed to the low concentration of yellow pigments in finer rice flour [30].

The particle size of rice flour significantly influences its functional attributes, such as water absorption, solubility, and textural characteristics. Small particles, with their large surface area, demonstrate improved water absorption and solubility, which are critical for various food manufacturing processes [31]. Given these properties, finer rice flour is suitable for applications requiring high viscosity and gel strength.

### 3.3. Textural Properties of Rice Flour

The influence of particle size on the textural properties of rice flour, including hardness, adhesiveness, cohesiveness, springiness, and gumminess, was analyzed in this study. The results demonstrated a consistent trend in which small particle sizes enhanced all measured textural properties (Table 3).

The hardness of rice flour increased significantly with the decrease in particle size, with values ranging from 8.20 ± 1.64 g for the 60-mesh sample to 30.66 ± 2.51 g for the 180-mesh sample. When comparing 60-mesh and 180-mesh samples, the hardness of the rice flour gel increased by approximately 3.7 times. This increase can be attributed to the large surface area of small particles, which enhances water absorption and strengthens the structural integrity of the flour matrix [32].

Adhesiveness also increased with the decrease in particle size, with values ranging from 1.33 ± 0.58 mJ for the 60-mesh sample to 2.20 ± 0.17 mJ for the 180-mesh sample. This trend is due to the enhanced interaction of fine particles within the batter matrix, which promotes strong bonding and adhesion. Additionally, this trend was consistent with a previous study by Lin et al. [27].

Cohesiveness improved significantly with the decrease in particle size, with values ranging from −0.01 ± 0.08 for the 60-mesh sample to 0.60 ± 0.08 for the 180-mesh sample. A fine particle size contributes to a uniform and stable internal structure, enhancing the overall cohesiveness of the rice flour matrix [22].

The springiness of rice flour also increased with smaller particle sizes, with values ranging from 6.75 ± 0.65 mm for the 60-mesh sample to 10.11 ± 0.60 mm for the 180-mesh sample. This improvement reflects the enhanced elasticity and hydration capacity of finer particles, which allow the flour to recover its original shape more effectively [33].

Furthermore, gumminess significantly increased from 0.33 ± 3.05 g for the 60-mesh sample to 11.33 ± 5.77 g for the 180-mesh sample. This trend can be attributed to the high density and compactness of the structure formed by small particles [34]. Smaller particles create a denser structure, which enhances the gumminess of the flour matrix.

### 3.4. Consistency of Rice Batter

**Figure 2:**
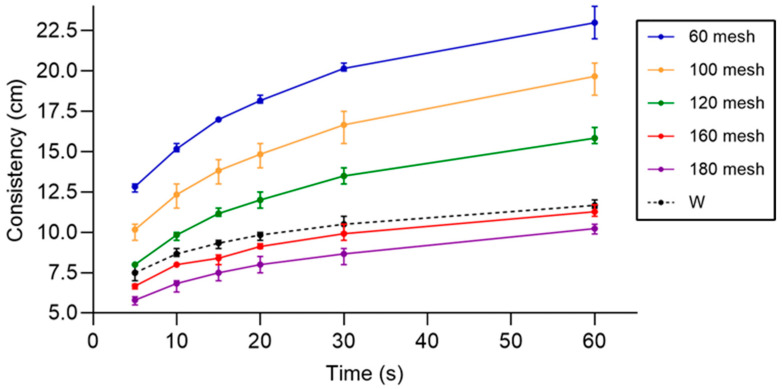
Consistency of Baromi-2 batter over time for different mesh sizes.

The particle size of Baromi-2 rice flour significantly influenced the consistency and rheological properties of the batter (Table 4 and Figure 2). Using a Bostwick viscometer, a larger particle size (e.g., 60 mesh) resulted in a long flow distance at all-time intervals, indicating low viscosity, whereas a smaller particle size (e.g., 180 mesh) demonstrated a short flow distance, reflecting high viscosity. For example, at 5 s, the batter with 60-mesh particles flowed to 12.83 cm, whereas the batter with 180-mesh particles flowed to 5.80 cm. By 60 s, these distances increased to 23.00 and 10.23 cm, respectively. This trend is depicted in Figure 2, which shows the progressive increase in flow distance over time for all particle sizes.

Figure 2 highlights the dynamic behavior of batter consistency, with larger particle sizes (e.g., 60 and 100 mesh) showing the steepest increase in flow distance, whereas finer particle sizes (e.g., 160 and 180 mesh) maintain a slower and more stable progression. Wheat flour batter (W), which was included as a control, demonstrates intermediate behavior, which is consistent with the 120-mesh rice flour batter. This visual comparison underscores the distinct influence of rice flour particle size on batter consistency and its time-dependent properties.

The enhanced viscosity observed in batters with a finer particle size is attributed to their high WHC. Smaller particles have a greater surface area and porosity, which promote water absorption and retention. This trend results in thicker, more viscous batters with reduced flowability. These findings are consistent with previous studies conducted by De La Hera et al. [12] and Matsunaga et al. [4], who showed that smaller particle sizes improve batter consistency and adhesion properties.

Furthermore, finer particle sizes improved coating stability during frying, resulting in more uniform coatings and reduced oil absorption. This result is consistent with the findings of Nakamura and Ohtsubo [5], who noted that smaller rice flour particles reduced oil uptake while maintaining a desirable texture in fried products. The data presented in Table 4 and Figure 2 indicate that particle size plays a critical role in optimizing batter functionality and achieving the desired rheological properties for fried food applications.

### 3.5. Physical Properties of Fried Chicken

The influence of particle size on the physical properties and crust color of the fried chicken was analyzed, particularly on pick-up percentage, cooking loss, moisture content, and crust color parameters (L*, a*, and b*). The pick-up percentage increased significantly as the particle size of the rice flour decreased, ranging from 6.55 ± 0.84% for the 60-mesh sample to 17.87 ± 1.47% for the 180-mesh sample (Table 5). The pickup rate of the 180-mesh sample was approximately 2.7 times higher than that of the 60-mesh sample. Therefore, finer particles enhance batter adhesion because of their large surface area and cohesive properties, thereby facilitating better binding to the chicken surface [5]. High pick-up percentages were positively correlated with the increased adhesiveness observed in the batter matrix. Notably, the pick-up percentage of 180-mesh Baromi-2 flour was comparable to that of wheat flour (17.23 ± 1.34%), indicating its potential application as a viable alternative to wheat flour in batter formulations.

Cooking loss decreased with the reduction in particle size, with the 60-mesh sample exhibiting the highest cooking loss at 34.26 ± 3.06%, whereas the 180-mesh sample showed a significantly lower cooking loss of 20.56 ± 0.57%. Cooking loss was reduced by approximately 40% in the 180-mesh sample compared to the 60-mesh sample. This reduction can be attributed to the ability of finer particles to form a dense and uniform batter matrix, which minimizes moisture evaporation during frying. This result is consistent with previous findings by Kyaw et al. [18], who demonstrated that thick and cohesive batter coatings effectively reduce cooking loss by limiting moisture escape and oil absorption [3].

The moisture content of the fried chicken increased with the decrease in particle size, ranging from 49.60 ± 0.24% for the 60-mesh sample to 61.18 ± 0.97% for the 180-mesh sample. This trend is attributed to the ability of finer particles to form a strong barrier effect in the batter, thereby effectively trapping moisture within the fried product [2]. The moisture content of the 180-mesh Baromi-2 flour was comparable to that of wheat flour-based batters (61.46 ± 2.38%), further supporting its application as a wheat flour alternative.

The crust color of the fried chicken also varied with particle size. The lightness (L*) value increased with finer particles, ranging from 47.99 ± 0.67 for the 60-mesh sample to 55.06 ± 0.14 for the 180-mesh sample. This trend indicates that finer particles produce a lighter crust color because of the uniform batter coverage and reduced browning during frying [4]. The redness (a*) value decreased from 8.07 ± 0.14 for the 60-mesh sample to 6.28 ± 0.17 for the 180-mesh sample, indicating a less intense red hue in finer particle samples. Similarly, the yellowness (b*) value slightly increased with finer particles, ranging from 23.73 ± 0.83 for the 60-mesh sample to 27.23 ± 0.10 for the 180-mesh sample, indicating that smaller particle sizes enhance the brightness and yellow hue of the crust [5]. Overall, finer rice flour particles improve the pick-up percentage, reduce the cooking loss, retain more moisture, and enhance the crust color of fried chicken. These properties highlight the potential application of finer rice flour particles, such as the 180-mesh Baromi-2 flour, in batter formulations to improve the quality and functionality of fried foods.

### 3.6. Textural Properties of Fried Chicken

Texture profile analysis of fried chicken made with Baromi-2 rice flour revealed notable trends in hardness, adhesiveness, cohesiveness, springiness, and chewiness, which are all influenced by the particle size of the flour (Table 6).

The hardness of the fried chicken decreased significantly with the decrease in the particle size of the flour, with the 60-mesh sample showing the highest hardness at 2252.80 ± 492.71 g, whereas the 180-mesh sample exhibited the lowest hardness at 1474.67 ± 267.97 g. As the rice flour particle size decreased from 60 mesh to 180 mesh, the hardness of fried chicken decreased by 34.6%. This trend indicates that smaller particles contribute to a softer texture in the fried product. The decrease in hardness can be attributed to the enhanced oil barrier effect of finer batter particles, which improves tenderness by retaining more moisture during frying [35].

Adhesiveness increased with the decrease in particle size, ranging from 0.80 ± 0.20 mJ for the 60-mesh sample to 2.25 ± 0.10 mJ for the 180-mesh sample. This enhancement is attributed to the improved interaction between finer batter particles and the chicken surface, resulting in strong adhesion properties. Enhanced adhesiveness facilitates better coating adherence and creates a uniform batter layer, which improves the overall frying performance by reducing cooking loss and enhancing crust integrity [35,36].

On the contrary, cohesiveness decreased with the increase in particle size, ranging from 0.74 ± 0.28 for the 60-mesh sample to 0.51 ± 0.34 for the 180-mesh sample. This reduction in cohesiveness indicates that finer particles create a less uniform batter matrix, making the fried product more prone to crumbling.

Springiness also decreased slightly with finer particles, ranging from 9.24 ± 0.64 mm for the 60-mesh sample to 7.46 ± 0.04 mm for the 180-mesh sample. This decrease in springiness indicates the soft texture of the fried chicken made with finer Baromi-2 particles.

Similarly to hardness, chewiness decreased from 207.90 ± 5.31 g for the 60-mesh sample to 54.85 ± 0.07 g for the 180-mesh sample. This decrease in chewiness reflects the soft and tender texture of the fried product as particle size decreases.

The texture profile of the fried chicken prepared with Baromi-2 rice flour approaches that of wheat flour (W), highlighting its potential as an effective alternative to wheat flour in batter formulations. These findings indicate the important effects of particle size on the textural properties of fried foods and confirm the role of adhesiveness in achieving optimal coating performance and batter stability [35,36].

## 4. Conclusions

This study underscores the important role of particle size in determining the rheological properties of batters and the quality attributes of deep-fat fried chicken. Baromi-2 rice flour, which was fractionated into varying particle sizes, demonstrated distinct functional and textural advantages based on its size distribution. A finer particle size (e.g., 180 mesh) not only exhibited higher WHC and batter viscosity compared to others, but also directly improved the coating pickup of the batter, moisture retention, and the texture of the final product. In particular, although finer particles tend to have a higher starch particle destruction rate, which may slightly increase the content of damaged starch, the damaged starch content of the 180-mesh rice flour used in this study (9.21%) was not excessive enough to impair the rheological properties of the batter. Rather, it is thought that the high moisture absorption capacity and viscosity formation contribution due to the increased amylose content and expanded particle surface area had a greater effect. These factors contributed to suppressing moisture loss during frying and inducing the formation of a clearer and denser crust, thereby implementing the desired texture. Apart from the improved textural and physical characteristics, finer particles produced light and visually appealing crusts, with high lightness (L*) and reduced redness (a*) and yellowness (b*). These attributes are consistent with consumer preferences for uniform and attractive fried products. Conversely, larger particle sizes (e.g., 60 mesh) were associated with a low viscosity and high cooking loss, resulting in thick and less adhesive batters, as well as diminished quality in the final fried product.

As the particle size of Baromi-2 rice flour decreased, it became more suitable as a wheat flour substitute in the batter system. The 120-mesh rice flour, which has a particle size similar to that of wheat flour, exhibited lower dough properties than wheat flour. However, as the particle size decreased, the 180-mesh sample improved the batter pickup (17.87%), cooking loss (20.56%), and moisture content (61.18%) to levels similar to those of the wheat flour chicken sample. Accordingly, the 180-mesh sample showed similar physical properties to wheat flour chicken, with hardness and springiness, which are important for the texture of fried food, reduced to 1474.67 g and 7.46 mm, respectively. This suggests that simply reducing the particle size can maintain the low oil absorption rate and excellent processability of Baromi-2 rice flour while achieving quality characteristics similar to wheat flour.

Sensory evaluation by trained panels will be necessary as the next step in this study. Moreover, future research should explore the interaction between rice flour and other functional ingredients, such as hydrocolloids, emulsifiers, and enzymes, to further enhance batter functionality. Scaling these findings to industrial production could expand the commercial application of Baromi-2 rice flour, providing a sustainable, high-quality option for modern food systems. The findings of this study provide insights into the strategic utilization of rice flour particle size, bridging the gap between consumer demand for healthier fried foods and the food industry’s need for cost-effective, versatile ingredients.

## Figures and Tables

**Table 1:** Particle-size distribution of Baromi-2 rice flour at different mesh sizes.

Size (Mesh)	Particle-Size Distributions			
	Mean (µm)	D10 (µm)	D50 (µm)	D90 (µm)
60	88.57 ± 0.17 ^a^	9.33 ± 0.11 ^a^	56.01 ± 1.17 ^a^	193.85 ± 0.71 ^a^
100	55.94 ± 0.37 ^b^	7.83 ± 0.83 ^b^	41.38 ± 0.74 ^b^	130.70 ± 0.00 ^b^
120	46.09 ± 0.44 ^c^	6.46 ± 0.42 ^c^	28.49 ± 0.60 ^c^	112.95 ± 1.06 ^c^
160	36.06 ± 0.47 ^d^	5.03 ± 0.10 ^d^	19.46 ± 0.32 ^d^	92.09 ± 1.02 ^d^
180	32.69 ± 0.95 ^e^	4.74 ± 0.03 ^e^	17.73 ± 0.35 ^e^	85.46 ± 2.35 ^d^

Values are presented as mean ± S.D. (*n* = 3). Different letters (a–e) in a column indicate significant differences (*p* < 0.05) by Duncan’s test.

**Table 2:** Physicochemical properties of rice flours with different particle sizes.

Size (Mesh)	Physicochemical Properties				Color		
	Moisture Content (%)	Water Holding Capacity (%)	Amylose Content (%)	Damaged Starch (%)	*L* *	*a* *	*b* *
60	10.66 ± 0.94 ^a^	1.11 ± 0.02 ^d^	20.33 ± 0.16 ^c^	4.45 ± 0.06 ^e^	92.96 ± 0.03 ^e^	−0.090 ± 0.01 ^b^	5.24 ± 0.01 ^a^
100	9.05 ± 0.62 ^b^	1.18 ± 0.01 ^c^	20.77 ± 0.35 ^bc^	6.49 ± 0.11 ^d^	93.26 ± 0.11 ^d^	−0.086 ± 0.01 ^b^	4.91 ± 0.05 ^b^
120	8.48 ± 1.06 ^bc^	1.25 ± 0.04 ^b^	21.14 ± 0.25 ^ab^	7.90 ± 0.21 ^c^	93.82 ± 0.10 ^c^	−0.083 ± 0.02 ^ab^	4.60 ± 0.25 ^c^
160	7.43 ± 0.22 ^c^	1.28 ± 0.03 ^b^	21.43 ± 0.09 ^a^	8.31 ± 0.01 ^b^	94.00 ± 0.07 ^b^	−0.076 ± 0.01 ^ab^	4.26 ± 0.01 ^d^
180	5.54 ± 0.43 ^d^	1.36 ± 0.02 ^a^	21.67 ± 0.23 ^a^	9.21 ± 0.01 ^a^	94.23 ± 0.02 ^a^	−0.057 ± 0.00 ^a^	4.14 ± 0.03 ^d^

This table shows the color properties (L*, a*, and b*) of rice flour with mesh sizes of 60, 100, 120, 160, and 180. Values are presented as mean ± S.D. (*n* = 3). Different letters (a–e) in a column indicate significant differences (*p* < 0.05) by Duncan’s test.

**Table 3:** Texture profile analysis of the batter prepared with rice flour of different particle sizes.

Size (Mesh)	Hardness (g)	Adhesiveness (mJ)	Cohesiveness	Springiness (mm)	Gumminess (g)
60	8.20 ± 1.64 ^e^	1.33 ± 0.58 ^c^	−0.01 ± 0.08 ^c^	6.75 ± 0.65 ^c^	0.33 ± 3.05 ^d^
100	14.00 ± 1.82 ^d^	1.53 ± 0.12 ^bc^	0.17 ± 0.11 ^bc^	7.79 ± 0.35 ^b^	2.33 ± 1.52 ^cd^
120	18.25 ± 2.75 ^c^	1.70 ± 0.17 ^b^	0.29 ± 0.15 ^b^	8.03 ± 0.78 ^b^	4.66 ± 2.51 ^bc^
160	23.00 ± 2.64 ^b^	1.96 ± 0.15 ^a^	0.36 ± 0.06 ^b^	9.25 ± 0.12 ^a^	7.33 ± 1.52 ^b^
180	30.66 ± 2.51 ^a^	2.20 ± 0.17 ^a^	0.60 ± 0.08 ^a^	10.11 ± 0.60 ^a^	11.33 ± 5.77 ^a^

Values are presented as mean ± S.D. (*n* = 3). Different letters (a–e) in a column indicate significant differences (*p* < 0.05) by Duncan’s test.

**Table 4:** Batter consistency (cm) of rice flour and wheat flour batters at different time intervals.

Size (Mesh)	Batter Consistency (cm)					
	5 s	10 s	15 s	20 s	30 s	60 s
60	12.83 ± 0.29 ^a^	15.17 ± 0.29 ^a^	17.00 ± 0.00 ^a^	18.17 ± 0.28 ^a^	20.17 ± 0.29 ^a^	23.00 ± 1.00 ^a^
100	10.17 ± 0.58 ^b^	12.33 ± 0.76 ^b^	13.83 ± 0.76 ^b^	14.83 ± 1.04 ^b^	16.67 ± 1.04 ^b^	19.67 ± 1.04 ^b^
120	8.00 ± 0.00 ^c^	9.83 ± 0.29 ^c^	11.17 ± 0.29 ^c^	12.00 ± 0.50 ^c^	13.50 ± 0.50 ^c^	15.83 ± 0.58 ^c^
160	6.67 ± 0.15 ^d^	8.00 ± 0.00 ^d^	8.40 ± 0.27 ^d^	9.13 ± 0.38 ^d^	9.92 ± 0.38 ^d^	11.28 ± 0.30 ^d^
180	5.80 ± 0.27 ^e^	6.83 ± 0.35 ^e^	7.50 ± 0.50 ^e^	8.00 ± 0.58 ^e^	8.67 ± 0.58 ^e^	10.23 ± 0.31 ^d^
W	7.50 ± 0.50	8.67 ± 0.29	9.33 ± 0.29	9.83 ± 0.29	10.50 ± 0.50	11.67 ± 0.29

W represents the wheat flour batter prepared in the same ratio as the Baromi-2 rice flour batter. Values are presented as mean ± S.D. (*n* = 3). Different letters (a–e) in a column indicate significant differences (*p* < 0.05) by Duncan’s test.

**Table 5:** Physical properties and crust color of fried chicken prepared with rice flour of different mesh sizes.

Size (Mesh)	Physical Properties			Color of Crust		
	Pick Up (%)	Cooking Loss (%)	Moisture Content (%)	L*	a*	b*
60	6.55 ± 0.84 ^e^	34.26 ± 3.06 ^a^	49.60 ± 0.24 ^c^	47.99 ± 0.67 ^d^	8.07 ± 0.14 ^a^	23.73 ± 0.83 ^d^
100	9.09 ± 0.99 ^d^	27.54 ± 1.27 ^b^	53.06 ± 2.77 ^bc^	51.23 ± 0.28 ^c^	7.70 ± 0.21 ^b^	25.08 ± 0.22 ^c^
120	11.37 ± 1.28 ^c^	24.17 ± 1.89 ^c^	53.97 ± 1.35 ^b^	51.71 ± 0.81 ^c^	7.16 ± 0.42 ^c^	26.28 ± 0.32 ^b^
160	14.86 ± 1.66 ^b^	22.27 ± 0.58 ^cd^	59.11 ± 0.01 ^a^	53.46 ± 1.02 ^b^	6.70 ± 0.17 ^d^	26.75 ± 0.34 ^ab^
180	17.87 ± 1.47 ^a^	20.56 ± 0.57 ^d^	61.18 ± 0.97 ^a^	55.06 ± 1.41 ^a^	6.28 ± 0.17 ^e^	27.23 ± 0.10 ^a^
W	17.23 ± 1.34	16.34 ± 0.25	61.46 ± 2.38	45.70 ± 0.42	5.86 ± 0.10	21.52 ± 0.24

W represents the wheat flour batter prepared in the same ratio as the Baromi-2 rice flour batter. Values are presented as mean ± S.D. (*n* = 3). Different letters (a–e) in a column indicate significant differences (*p* < 0.05) by Duncan’s test.

**Table 6:** Texture profile analysis of Baromi-2 rice flour and wheat flour batters by particle size.

Size (Mesh)	Hardness (g)	Adhesiveness (mJ)	Cohesiveness	Springiness (mm)	Chewiness (g)
60	2252.80 ± 492.71 ^a^	0.80 ± 0.20 ^d^	0.74 ± 0.28 ^a^	9.24 ± 0.64 ^a^	207.90 ± 5.31 ^a^
100	2020.67 ± 349.07 ^ab^	1.23 ± 0.25 ^c^	0.62 ± 0.53 ^b^	8.49 ± 0.29 ^b^	143.55 ± 24.11 ^b^
120	1751.33 ± 514.80 ^ab^	1.50 ± 0.10 ^bc^	0.59 ± 0.43 ^bc^	8.24 ± 0.12 ^b^	100.05 ± 51.12 ^c^
160	1627.00 ± 503.46 ^ab^	1.73 ± 0.21 ^b^	0.56 ± 0.25 ^cd^	7.70 ± 0.16 ^c^	58.77 ± 5.96 ^cd^
180	1474.67 ± 267.97 ^b^	2.25 ± 0.10 ^a^	0.51 ± 0.34 ^d^	7.46 ± 0.04 ^c^	54.85 ± 0.07 ^d^
W	1490.75 ± 300.68	1.65 ± 0.34	0.60 ± 0.03	7.75 ± 0.29	70.87 ± 13.02

W represents the wheat flour batter prepared in the same ratio as the Baromi-2 rice flour batter. Values are presented as mean ± S.D. (*n* = 3). Different letters (a–d) in a column indicate significant differences (*p* < 0.05) by Duncan’s test.

## Data Availability

The original contributions presented in this study are included in the article. Further inquiries can be directed to the corresponding author.
